# Gut microbiota composition and metabolomic profiles of wild and captive Chinese monals (*Lophophorus lhuysii*)

**DOI:** 10.1186/s12983-020-00381-x

**Published:** 2020-12-03

**Authors:** Dandan Jiang, Xin He, Marc Valitutto, Li Chen, Qin Xu, Ying Yao, Rong Hou, Hairui Wang

**Affiliations:** 1 Chengdu Research Base of Giant Panda Breeding, Chengdu, 610081 China; 2 Sichuan Key Laboratory of Conservation Biology for Endangered Wildlife, Chengdu, 610081 China; 3 Sichuan Academy of Giant Panda, Chengdu, 610081 China; 4 EcoHealth Alliance, New York, NY 10012 USA; 5 Sichuan Fengtongzhai National Nature reserve administration, Yaan, 625700 China

**Keywords:** Chinese monal, Metabolite, Gut microbiota, Feces

## Abstract

**Background:**

The Chinese monal (*Lophophorus lhuysii*) is an endangered bird species, with a wild population restricted to the mountains in southwest China, and only one known captive population in the world. We investigated the fecal microbiota and metabolome of wild and captive Chinese monals to explore differences and similarities in nutritional status and digestive characteristics. An integrated approach combining 16S ribosomal RNA (16S rRNA) gene sequencing and ultra-high performance liquid chromatography (UHPLC) based metabolomics were used to examine the fecal microbiota composition and the metabolomic profile of Chinese monals.

**Results:**

The results showed that the alpha diversity of gut microbes in the wild group were significantly higher than that in the captive group and the core bacterial taxa in the two groups showed remarkable differences at phylum, class, order, and family levels. Metabolomic profiling also revealed differences, mainly related to galactose, starch and sucrose metabolism, fatty acid, bile acid biosynthesis and bile secretion. Furthermore, strong correlations between metabolite types and bacterial genus were detected.

**Conclusions:**

There were remarkable differences in the gut microbiota composition and metabolomic profile between wild and captive Chinese monals. This study has established a baseline for a normal gut microbiota and metabolomic profile for wild Chinese monals, thus allowing us to evaluate if differences seen in captive organisms have an impact on their overall health and reproduction.

## Background

The Chinese monal (*Lophophorus lhuysii*) belongs to the order Galliformes, family Phasianidae, distributed in the mountains of southwest China at an elevation of 3000 to 4900 m [1]. It is an endemic bird species of China, which has been listed as endangered by the Convention on International Trade in Endangered Species of Wild Fauna and Flora (CITES) [2], classified as a vulnerable species on the International Union for Conservation of Nature (IUCN) red list [3]. There has been limited success with establishing captive breeding groups of Chinese monals throughout the world, the reason for which is not entirely clear [4]. At present, the world’s only captive collection exists in the Fengtongzhai National Nature Reserve in Sichuan, China, with only 23 individuals as of early 2020. The IUCN states the wild population of Chinese monal continues to decrease with research needed to understand their ecology and threats to their livelihood (Available at https://www.iucnredlist.org/species/22679192/30181918). However, given the limited access to the species and their biological samples, few studies have been conducted in captive and wild Chinese monals [4]. With a declining population, it is imperative to learn more about the Chinese monal to ensure a healthy existence of the species.

The gut microbiota is recognized as a coevolutionary partner with an ecosystem that is metabolically adaptable, rapidly renewable, and metabolically flexible [5, 6]. It performs numerous beneficial functions for the host, such as nutrient acquisition, immunomodulation and physiogenesis in response to profound lifestyle changes [7, 8]. The composition and diversity of gut microbiota is influenced by numerous factors, such as diet composition [9], social interactions [10] and health status [11]. For example, wildlife that is maintained in a captive setting have significant differences in diet, social structure, and stress, all of which potentially affect their gut microbiota. Previous studies have demonstrated that captivity affects the composition of gut microbes in birds [11–14] and mammals [9, 10, 15, 16], with considerable differences were observed between wild and captive individuals. Thus, study on the gut microbiota in both wild and captive animals may provide a way to understand more about how to successfully maintain captive wildlife and in optimal health condition.

The fecal metabolomic profile is the product of functional activity of both host cells and gut bacteria, which provides information on their combined past activity [17]. Any differences in the microbial community may have a significant effect on the metabolite profiles of the host [18], which can be explored through untargeted metabolomics. The combined study of fecal 16S ribosomal RNA (16S rRNA) gene sequencing and untargeted metabolomics is expected to uncover the inherent associations between the microbiota and the host metabolic phenotypes [18], and have been used to elucidate the differences in gut microbes and host metabolic phenotypes in the context of different food composition [19, 20].

Given the critical role of gut microbiota to the host and its close relationship with the host’s metabolic phenotype, the study of gut microbial composition and metabolomic profile could be helpful to understand the health and nutritional status of the captive Chinese monal (CCM) in relation to the wild Chinese monal (WCM). Therefore, the objectives of the present study were to investigate the fecal microbiota and metabolome of WCM and CCM, and discuss the causes for the differences. The study may provide a theoretical basis for the future breeding of CCM, a species whose wild population is declining and for which there are limited scientific publications regarding species health.

## Results

### Monal species identification

There were six and 35 samples of the feces in Yuancaopo and Hongshanding belonging to the Chinese monal, respectively, according to the species identification. The remaining samples were identified as 24 blood pheasants (*Ithaginis cruentus*), six temminck’s tragopan (*Tragopan temminckii*), and 20 samples without amplification results.

### Microbial community composition

**Fig. 1:**
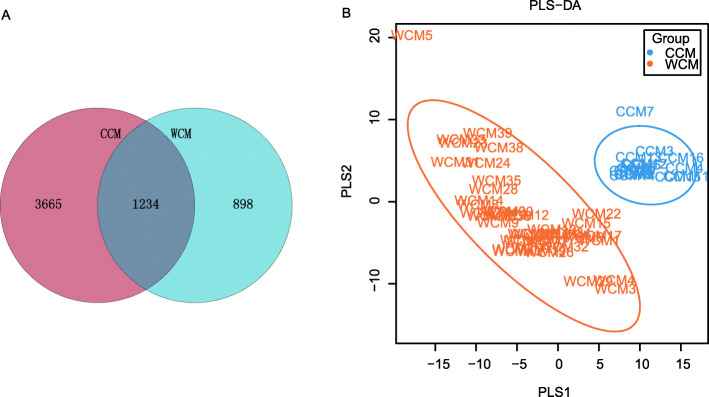
Venn plot (**a**) and Partial least squares discrimination analysis (PLS-DA) scores scatter plot (**b**) of fecal microbiota of captive (CCM) and wild Chinese monal (WCM)

A total of 1,466,455 high-quality reads with an average sequencing depth of 35,767 ± 1265 reads per WCM fecal sample was conducted and classified into 2132 operational taxonomic units (OTUs). Whereas, 580,736 high-quality reads with an average sequencing depth of 36,296 ± 1062 reads per CCM fecal sample was conducted and classified into 4899 OTUs. The number of OTUs present in both the wild and captive groups was 1234, with 898 unique OTUs in the wild group, and 3665 unique OTUs in the captive group (Fig. 1a). The rarefaction curves (Fig. S1A) and rank abundance curves (Fig. S1B) of the WCM and CCM fecal samples convey the richness and evenness of the microbial species in the samples, and the rarefaction curves had reached a plateau. These findings demonstrated that the sequencing data was reasonable and reflected the number of samples in this study was sufficient.

The gut microbiota alpha diversity indexes (Shannon and Chao1) in WCM were significantly higher than that of the CCM at the microbial species level (Kruskal-Wallis test: Effect sizes = 0.137, 0.358; *P* = 0.006, 7.63E-06. Wilcoxon test: W = 484, 580; *P* = 0.006, 1.299E-06). The discrepancy between the WCM and CCM was further analyzed via the partial least squares discrimination analysis (PLS-DA) plot as illustrated in Fig. 1b. The distance between the orange and blue color markers demonstrated a unique bacterial community structure in WCM vs. CCM, suggesting that there was a significant difference in gut microbial composition between the two groups.

**Fig. 2:**
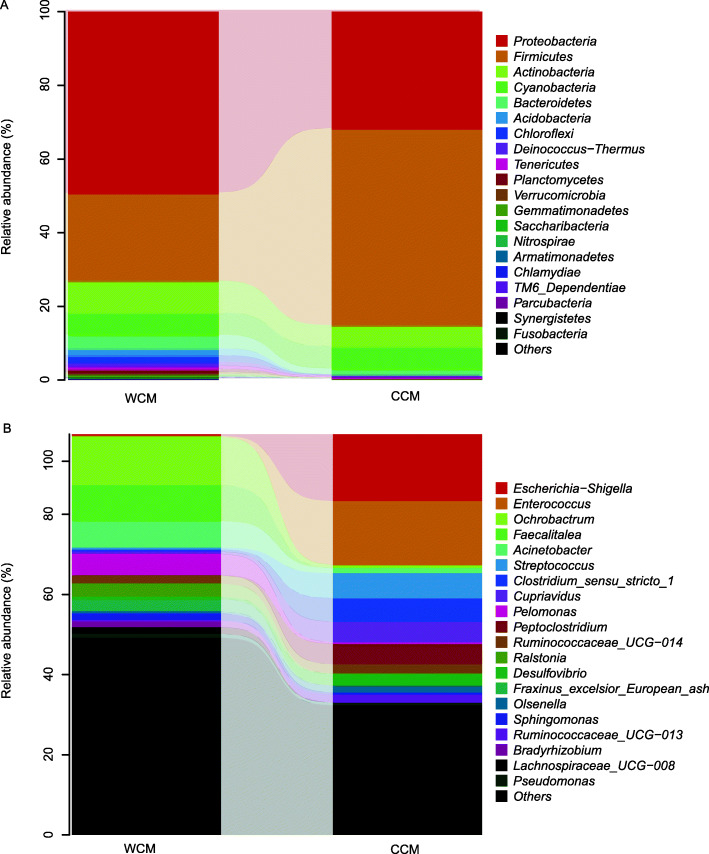
The fecal microbiota composition profiles at the phylum (**a**) and genus (**b**) level in the captive (CCM) and wild Chinese monal (WCM)

The predominant microbial phyla identified included *Proteobacteria* (49.6%), *Firmicutes* (23.8%), *Actinobacteria* (8.7%) in the wild group, and *Firmicutes* (53.5%), *Proteobacteria* (32.0%), *Cyanobacteria* (6.0%) in the captive group. At the genus level, *Ochrobactrum* (12.1%) dominated the WCM gut microbiota, followed by *Faecalitalea* (9.0%), and *Acinetobacter* (6.7%), whereas, for captive individuals, *Escherichia-Shigella* (16.7%), *Enterococcus* (16.0%) and *Streptococcus* (6.3%) were primarily identified. The top 20 species for each group at phylum and genus levels were shown in Fig. 2a and b, respectively.

### Metabolomic profiles

**Fig. 3:**
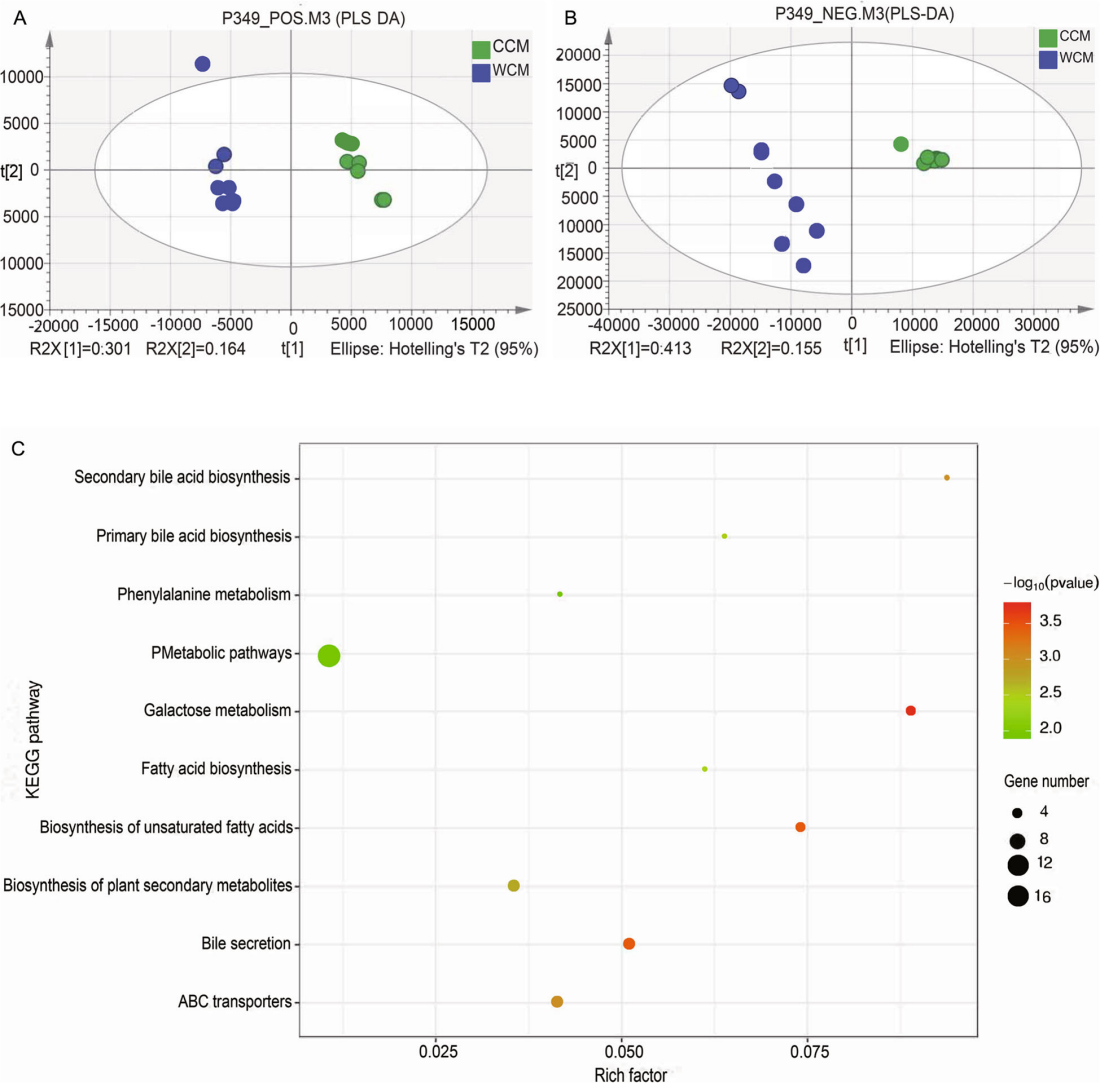
Partial least squares discrimination analysis (PLS-DA) score plots of fecal samples from the captive (CCM) and wild Chinese monal (WCM) group in positive (**a**) and negative (**b**) ion modes. KEGG enrichment analysis bubble map display the most significant top 10 enrichment metabolic pathway (**c**). Rich factor refers to the ratio of the number of significantly different metabolites detected to the number of metabolites annotated in the pathway, and the higher value of rich factor represents greater enrichment. The size of the point represents the enrichment of significant metabolites in the corresponding metabolic pathway

**Fig. 4:**
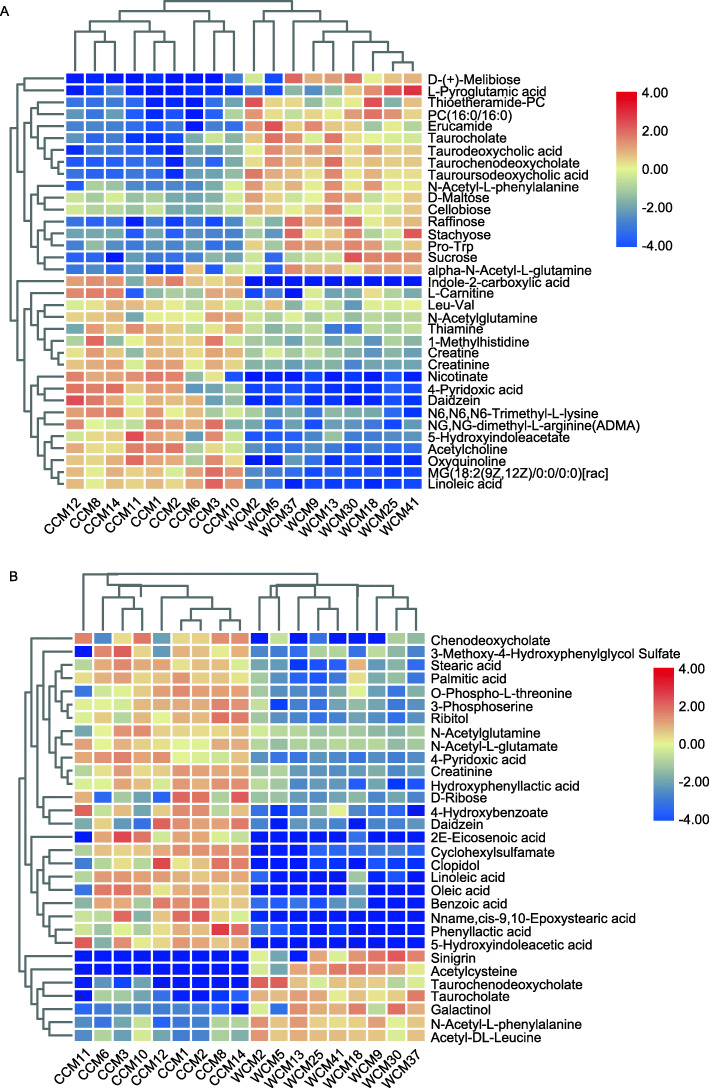
Heatmap summarizing fold changes of significantly altered metabolites in the sequencing data of fecal samples in the positive (**a**) and negative (**b**) modes, respectively. Red and blue represent higher and lower concentrations of metabolites in the corresponding abscissa samples, respectively

Metabolites in Chinese monal fecal samples were acquired in both positive (POS) and negative (NEG) ion modes. A total of 41,943 mass spectral features were detected, of these, 146 and 177 metabolites were positively identified in POS and NEG modes, respectively, from a library of 2500 known biochemical compounds. PLS-DA score plots showed a distinctive difference in the metabolic profile between the two groups (Fig. 3a & b). In the POS and NEG modes, 58 significant metabolites were identified whose VIP-value>1 and *P*-value ≤0.05. The heatmap of Hierarchical Clustering was shown in Fig. 4a, b. These metabolites were mainly grouped into fatty acids, bile acids derivatives, sugars, and indole derivatives. These metabolites were then submitted to the Kyoto Encyclopedia of Genes and Genomes (KEGG) website for relevant pathway analysis, the results of which showed that these metabolites were associated with 20 significant enrichment KEGG pathways. The top 10 most significant enrichment results are shown in Fig. 3c. These pathways were primarily related to galactose metabolism, starch and sucrose metabolism, metabolic pathways, biosynthesis of fatty acid, biosynthesis of bile acid, and bile secretion.

### Microbiota–metabolome association

**Fig. 5:**
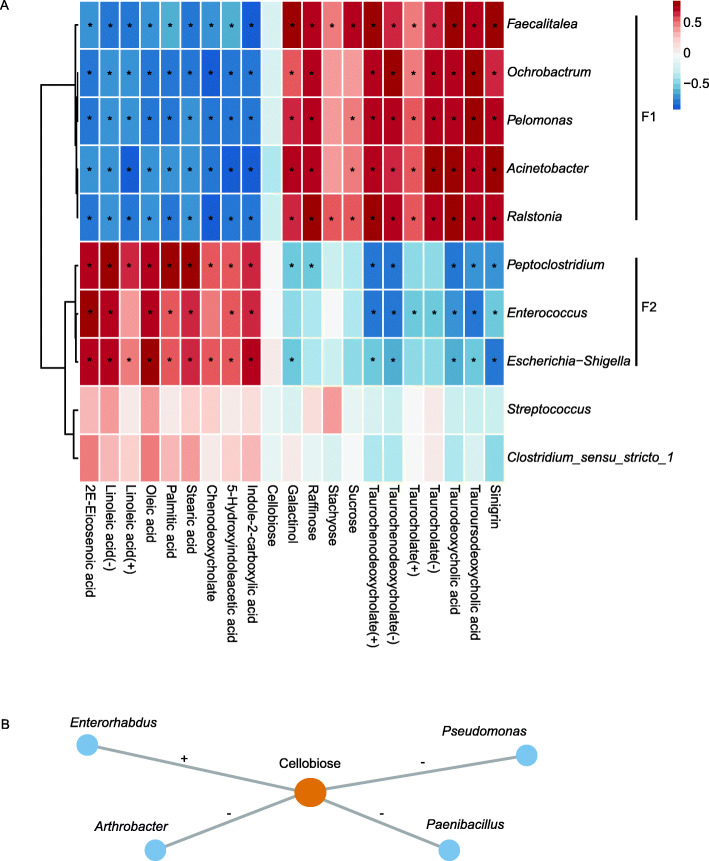
Correlation plot showing the functional correlation between the top 10 most common fecal bacteria genera and specific fecal metabolites (**a**), and significant correlations between cellobiose and the detected genus (**b**). Red and blue square represent the positive and negative correlations between metabolites and bacteria, respectively. (+), (−) denote metabolites detected in positive and negative ion modes, respectively. *, +, − all represent *p* < 0.05, |*r*| > 0.5, this means that there are significant correlations between bacteria and metabolites

Strong associations between microbiota composition (at genus level) and metabolite perturbations were revealed by a Mantel test (*r* = 0.825, *P* = 0.001). Significant correlations could be detected between the fecal microbiota and metabolite based on Spearman correlation coefficients (*r* > 0.5 or *r* < − 0.5, *P* < 0.05) [21]. Among the metabolites, fatty acids, bile acid derivatives, and carbohydrate were involved in more than half of the metabolic pathways. Metabolites that were remarkably different were highly correlated with microorganisms. The correlations between specific fecal metabolites and the top 10 most common fecal bacteria genera were shown in Fig. 5a.

*Ochrobactrum*, *Faecalitalea*, *Acinetobacter*, *Pelomonas*, *Ralstonia* were the dominant genera of the wild group, represented by F1, *Escherichia-Shigella*, *Enterococcus*, *Peptoclostridium*, *Streptococcus* and *Clostridium_sensu_stricto_1* were dominant in the captive individuals, and *Escherichia-Shigella*, *Enterococcus* and *Peptoclostridium* were represented by F2. The results showed fatty acids (linoleic acid, oleic acid, palmitic acid, stearic acid, 2E-eicosenoic acid), and bile salt (chenodeoxycholate) were higher in the feces of the CCM and negatively correlated with F1, but mainly positively correlated with F2. However, carbohydrate (stachyose, galactinol, sucrose, and raffinose), bile acid (taurodeoxycholic acid, tauroursodeoxycholic acid) and bile salts (taurochenodeoxycholate, taurocholate) were higher in the feces of WCM and mainly negatively correlated with F2, but positively correlated with F1. Remarkably, 5-hydroxyindoleacetic acid and indole-2-carboxylic acid, which increased 113.3 and 295.7-fold, respectively, in CCM, negatively correlated with F1 but positively correlated with F2. Moreover, Sinigrin was decreased 3189.2-fold in the CCM, and positively correlated with F1 but negatively correlated with F2. There was no significant correlation between cellobiose and F1, F2 (Fig. 5a), while cellobiose was positively correlated with *Enterorhabdus* and negatively correlated with *Arthrobacter*, *Pseudomonas* and *Paenibacillus* (Fig. 5b).

## Discussion

Through fecal analysis, we studied the gut microbiota structure and the metabolomic profiles in both WCM and CCM. The results of this research indicated distinct differences of the fecal bacterial communities between the two groups, furthermore, we identified significant correlations between the fecal microbiota and metabolites.

The levels of alpha and beta-diversity of microbiota from WCM were significantly higher than CCM, indicating a more diverse microbial community in the wild population. These differences may be the result of differences in diet composition, geographical ranges, energy utilization, climate conditions, and stress exposure in the WCM vs. the CCM [10, 22].

*Proteobacteria* was the dominant phylum in the feces of WCM followed by *Firmicutes*, while *Firmicutes* was relatively more abundant than *Proteobacteria* in the feces of the captive group. The characterization of gut bacteria in several studies reveals a dominance of *Firmicutes* and *Bacteroidetes*, which appears to be a common feature of birds. The dominance of these phyla has been described in the ceca of turkeys (*Meleagris gallopavo*) [23], the cloacal samples of Chinstrap penguins (*Pygoscelis antarctica*) [24], the feces of Japanese quail (*Coturnix japonica*) [25], as well as in the crop of hoatzin (*Opisthocomus hoazin*) [26]. However, previous studies also reported that *Proteobacteria* and *Firmicutes* are the dominant phyla in the feces of macaroni penguins (*Eudyptes chrysolophus*), little blue penguins (*Eudyptula minor*) [27], *Procellariiform* seabirds [28], Lady Amherst’s pheasant (*Chrysolophus amherstiae*), Reeves’s pheasant (*Syrmaticus reevesii*), and Cabot’s tragopan (*Tragopan caboti*) [29], which is more consistent with our observations of the microbiota composition in the Chinese monal. Moreover, *Bacteroidetes* just accounted for 3.8 and 1.6%, respectively, in the WCM and CCM unlike what was observed in the turkey, Chinstrap penguin, quail, and hoatzin studies. Based on the results of this study, it would appear that the gut microbial composition of the Chinese monal is most reflective of that which has been detected in other species of the family Phasianidae; however, in the aforementioned cited research only two samples per group were collected and study details are lacking which highlights the need for further research on this subject.

A large diversity of gut microbes has been described as adaptive and beneficial, with diet being considered one of the most critical factors shaping gut microbial structures. The Chinese monal is an omnivorous bird. For wild populations, food is scarce in the winter months, due to their natural environment being at a high altitude and low temperatures. Whereas, the small captive population of monals receives a relatively steady diet with no change in environment except for exposure to outdoor seasonal climate conditions. Our results indicated a high abundance of *Proteobacteria* in WCM. *Proteobacteria* have greatly variable morphology and versatile functions [30], previous studies have demonstrated that an increased richness of *Proteobacteria* in the gut microbial flora is mainly related to energy accumulation [31–33], and is more abundant when the host animal has prolonged exposure to cold climates [32]. We suspect our findings in WCM may be in response to their comparatively complex dietary composition, as well as an increase in energy storage in preparation for cold weather and a reduction in food availability.

In contrast to WCM, *Firmicutes* was identified as the dominant bacteria phylum in CCM. Compared with the WCM, we suspect captive individuals are fed a diet with a comparatively higher lipid content. Diet composition, as indicated in Table 1 showed a 6.2% crude fat in the commercial pelleted feed, which provides the base of the diet, in addition to corn which contains 3.6 to 5.3% crude fat (The data provided at the China Feed-database Information Network Centre: http://www.chinafeeddata.org.cn/picture/pdf/CFIC2019_1.pdf). In a 2014 study, mice were fed a high-fat diet, and the *Firmicutes: Bacteroidetes* ratio in mice gut microbiota increased after 3 weeks, with the abundance of *Firmicutes* increased and *Bacteroidetes* depleted [34]. This finding is in accordance with many former studies, suggesting that a high-fat content in diet is one of the primary factors responsible for changes observed in gut microbe composition [7, 35]. Thus, the increased abundances of *Firmicutes* and the *Firmicutes*: *Bacteroidetes* ratio in CCW group (CCM 33.45 versus WCM 6.25) is likely the result of a higher lipid content in the diet.

**Table 1:** Nutrient levels of commercial pellet feed (as fed basis, %)

Component	Water	Crude fat	Crude fiber	Crude protein
**%**	11.1	6.2	2.9	17.4

Concerning the metabolome, we detected 58 significant metabolites that associated with 20 metabolic pathways. We found that a significantly higher fatty-acid content in feces of the CCM was related to unsaturated fatty acid and fatty acid biosynthesis. A previous study found that different dietary triacylglyceride composition has an influence on fatty acid content in the feces of human neonates [36]. Thus, our finding may be related to the nutrient composition of the diet, which contains a higher level of fat and a different lipid composition than what wild specimens are likely consuming. Otherwise, our results showed that the primary microbial genera in Chinese monal feces are significantly related to the metabolites we identified; therefore, microbial metabolism is an essential aspect that needs to be evaluated. Microbial biosynthesis of fatty acid may be due to cellular structure or storage, de novo synthesis from glucose, or incorporation of exogenous fatty acids directly into lipid structures [37, 38].

The content of metabolites in the feces relating to bile acid biosynthesis and bile secretion had a statistically significant difference between the WCM and CCM, including chenodeoxycholate, taurochenodeoxycholate, taurocholate, taurodeoxycholic acid, and tauroursodeoxycholic acid. Our results showed differences in metabolism of bile acid, an important component of bile, which plays a role in digestion and metabolism of dietary lipids and cholesterol [39, 40]. Chenodeoxycholate, taurochenodeoxycholate, and taurocholate are bile salts that are hydrolyzed into free bile acids by bile salt hydrolase [41]. Chenodeoxycholate amount was higher in the feces of CCM, whereas taurochenodeoxycholate and taurocholate were more abundant in the WCM. Taurodeoxycholic acid and tauroursodeoxycholic acid are taurine conjugated bile acids that were expectedly more abundant in the feces of WCM. The type of bile salt that is most abundant may reflect how the corresponding bile acids play a major role in digestion which is dependent on the dietary composition of each individual bird [42]. In the wild, Chinese monals primarily consume seeds and roots of shrubs in the winter when food is scarce, but they also consume a small amount of moss, earthworms and insect pupa [43, 44]. High concentrations of total non-structural carbohydrate (TNC) reserves are usually found in root tissues of plants [45–47], and may serves as one of the main energy sources for WCM. Because of the seed consumption, the WCM also need to secrete bile acids for fat digestion thus providing another valuable energy source. We also found a high correlation between the bile acid metabolites and gut microbial flora. There exists a mutual regulation between intestinal microbes and bile acids, for bile acid composition seems to be a critical regulator of microbiota structure, which in turn plays a key role in regulation of bile acid pool size, composition and metabolism [48, 49]. Kim G et al. [41] found that several bacterial species of *Firmicutes* and *Actinobacteria* can hydrolyze bile salt, among which *Enterococcus* was significantly related to the above metabolites in this study. A significantly higher abundance of carbohydrates was found in the feces of WCM as opposed to the CCM, including galactinol, raffinose, stachyose, sucrose, and cellobiose. In plants, raffinose and stachyose are oligosaccharides synthesized from sucrose, subsequently galactitol and galactose are also involved [50]. Animals don’t secrete enzymes that utilize raffinose-series oligosaccharides, which are likely to be digested by microbial enzymes at the end of the gastrointestinal tract [51]. Coon et al. [52] reported that ileal digestibility of raffinose and stachyose was less than 1.0% in roosters; however, the digestibility, determined with excreta collection, reached 90.5 and 83.8%, respectively. Previous studies have also indicated several Passerine bird species have a low digestibility of sucrose [53, 54] as fecal sugar content increased after consuming sucrose solutions [54]. Cellobiose is an important hydrolytic product of cellulose degradation [55]. Plants contain significant quantities of cellulose, which is difficult to naturally digest by most birds species, but can be utilized by bacteria inhabiting the intestinal tract, especially within the ceca [56]. Yuzhang Wang [57] reported that the cecum of the Chinese monal is well developed, accounting for 24.09% of the total length of the intestine, which may help the species utilize cellulose and other indigestible components of the diet. Microorganisms in genus level that have significant positive correlation with these metabolites may play a big role in these processes.

Indole-containing metabolites were significantly different in fecal samples of CCM, such as 5-hydroxyindoleacetic acid (5-HIAA) and indole-2-carboxylic acid, which are nearly 100 and 300 times higher than that in the wild, respectively. Indole and its derivatives widely exist in animals, microorganisms, and plants [58], and are the main metabolites of serotonin [59]. Degg et al. [60] found that excretion of indole containing metabolites, such as 5-HIAA, increased after humans ingested high serotonin-containing foods including tomatoes, bananas, etc. Our results which indicate higher levels of 5-HIAA and indole-2-carboxylic acid in CCM feces is likely associated with the incorporation of tomatoes in their routine diet. These two altered indole-containing metabolites were also highly correlated with fecal microbial flora. Previous studies have suggested that intestinal bacteria can convert tryptophan to indole through enzymatic processing, and then form indole-containing metabolites [40, 61]. These studies showed that metabolic processes in intestinal bacteria are essential for the synthesis of indole-containing metabolites, therefore, it may be a specific indicator of intestinal bacterial differences.

Finally, the last metabolite with a significant difference observed between the two groups is sinigrin (2-propenyl glucosinolate). Sinigrin was several thousand times higher in the feces of WCM than that observed in CCM. Sinigrin, which is enriched in plants such as cruciferous vegetables, leaf mustard, and horseradish, has been associated with carbohydrate regulation and lipid metabolism and has also been shown to have anti-neoplasia and anti-microbial properties [62]. In vitro experiments confirmed that sinigrin was degraded by rat intestinal microbiota [58]. *Enterococcus* and *Bacteroides* in the human intestine can degrade glucosinolates [63], and have a significant positive correlation with sinigrin in this study. Therefore, based on our findings of high levels of sinigrin in WCM feces, we can speculate that the diet of WCM also contains a large amount of sinigrin. Although the intestinal flora plays a role in the digestion process, sinigrin is more excreted through feces.

## Conclusion

This is the first study to evaluate the fecal microbiota and the fecal metabolic profiles in the Chinese monal. The results clearly demonstrate the differences of the gut bacterial composition and metabolism of the host and microbiota between wild and captive Chinese monals. The results of this study, will without a doubt, enhance our knowledge of wild and captive Chinese monals which may help guide recommendations that will affect the health and fecundity of birds in the captive breeding program. Further studies are required to determine if there is a difference in the fecal microbiota and metabolic profiles in spring and summer months when the dietary composition is likely to change.

## Methods

### Birds selected, study site and sample collection

Samples were collected from October to December of 2018 at Baoxing Fengtongzhai National Nature Reserve in Sichuan Province, China. Fecal samples from the wild were collected at two research sites, located on two mountains: Yuancaopo (30°34′38′′-30°35′3′′N, 102°48′15′′- 102°49′57′′E) and Hongshanding (30°37′39′′- 30°37′46′′N, 102°54′57′′- 102°55′3′′E). Samples that appeared fresh (e.g. still wet) were collected at an altitude of 3223.7 to 3568.7 m, and a temperature of − 17 °C – 5 °C. In Yuancaopo and Hongshanding, 49 and 42 samples were collected, respectively.

In November 2018, fecal samples of 11 captive birds (3 males and 8 females) were collected at the Chinese Monal Conservation and Research Center, where the altitude is 1582.0 m and the temperature range from 9 °C – 13 °C. These monals are maintained in four adjacent enclosures, with three enclosures containing one male each paired with one or two females, while one enclosure contains only three sub-adult females. Feces was collected by placing clean cardboard beneath the nightly roosting perches, overnight. The following morning at 8 AM, four fecal samples per enclosure were collected from the cardboard with an attempt to collect feces with varying colors and shapes.

Approximately 1 g of fecal material was collected from the central section of each fecal dropping, from both wild and captive animals, and placed into 1.5 mL sterile collection tubes (Corning, New York, USA) and immediately frozen in liquid nitrogen. Thereafter, the samples were shipped to the Chengdu Research Base of Giant panda Breeding within 24 h., where they were stored at − 80 °C for subsequent analysis.

### Measurement of the main components of the commercial pellet feed

The commercial pellet (New hope group, Chengdu, China) being fed to the CCM was analyzed to measure the primary nutritional components according to Chinese national standards. Moisture was determined by direct drying method [64]. Crude fat was determined by petroleum ether extraction method [65]. Crude protein was determined by Kjeldahl method [66]. Crude fiber was determined by Soxhlet extraction method [67].

### Monal species identification

Genomic DNA was isolated from the fecal samples using the TIANamp Micro DNA Kit (Tiangen, Beijing, China) following the manufacturer’s instructions. The DNA was amplified using cytochrome *b* (Cyt *b*) gene primers; forward: 5′-ACATTGGACGCGGCCTCTAC-3′ and reverse: 5′-GTGGGCGAAATGTTATGGTT-3′. PCR products were separated by electrophoresis in a 1% agarose gel, then recovered using the AxyPrep DNA gel recovery kit (Axygen, California, USA). The purified PCR products were sequenced using an ABI3730-XL genetic sequencer (Applied Biosystems, Foster City, CA, USA). Genetic sequencing data were blasted against the nucleic acid database of the National Center for Biotechnology Information (NCBI) using the Basic Local Alignment Search Tool (BLAST) program. Species identification was selected based on results that returned a BLANK % sequence similarity result.

### 16S rRNA microbial community analysis

The samples were homogenized separately, and then the total DNA was extracted from fecal samples (200 mg per sample) of all Chinese monal using the DNeasy PowerSoil Kit (QIAGEN, Inc. Netherlands), using their designated protocol. The V4-V5 region of the 16S rRNA gene was PCR amplified using the forward primer 515 F: 5′-GTGCCAGCMGCCGCGGTAA-3′ and the reverse primer 907 R: 5′-CCGTCAATTCMTTTRAGTTT-3′. Sample-specific 7-bp barcodes were incorporated into the primers for multiplex sequencing. Before being pooled, PCR amplicons were purified with Agencourt AMPure Beads (Beckman Coulter, Indianapolis, IN, USA), quantified using the PicoGreen dsDNA Assay Kit (Invitrogen, Carlsbad, CA, USA), and finally, paired-end sequencing with 2 × 300 bp read lengths was performed using the Illumina MiSeq platform with MiSeq Reagent Kit v3 at Shanghai Personal Biotechnology Co., Ltd. (Shanghai, China).

The raw sequencing data were processed using the Quantitative Insights Into Microbial Ecology (QIIME, v1.8.0) software package. Briefly, raw sequencing reads with exact matches to the barcodes were assigned to respective samples and identified as valid sequences. The low-quality sequences were filtered through criteria as previously described: sequences that had a length of < 150 bp, sequences that had average Phred scores of < 20, sequences that contained ambiguous bases, and sequences that contained mononucleotide repeats of > 8 bp [68]. Potential chimreas were removed using the Flash usearch61 tool with the minsnp parameter set to 2, and the remaining high-quality sequences were clustered into OTUs with a threshold of 97% sequence similarity by UCLUST. OTUs containing less than 0.001% of total sequences across all samples were discarded, and OTU taxonomic classification was conducted via BLAST searching the representative sequences set against the Greengenes Database [69] using the best hit [70].

Sequence data analyses were primarily calculated using QIIME (v1.8.0) and R packages (v3.2.0). We generated OTU-level ranked abundance curves with R package “ggplot 2” to compare the richness and evenness of OTUs among samples. Venn diagram was obtained to visualize the mutual and unique OTUs between groups using R package “VennDiagram”. Alpha diversity was calculated with QIIME software and discrepancies analyzed between the two groups using the Kruskal-Wallis test and Wilcoxon test with R software. Beta diversity analysis was performed to investigate the structural variation of microbial communities across samples using UniFrac distance metrics [71] and visualized via PLS-DA using the mixOmics package in R software. The microbiota compositional profiles could be achieved at different taxonomic levels, including phylum, class, order, family and genus, wherewith the taxa abundances at these levels were statistically compared among samples or groups by Metastats [72]. The sequences data available for our study were submitted to NCBI Sequence Read Archive under the BioProject ID PRJNA662165.

### Metabolomic analyses

Nine samples were selected from WCM and CCM, respectively, for untargeted metabolomics analyses, which was performed using ultra-high performance liquid chromatography (UHPLC) (1290 Infinity LC, Agilent Technologies, Palo Alto, USA) coupled to a Triple TOF 6600 mass spectrometer (AB Sciex, Foster City, USA) equipped with an electrospray ionization (ESI) source in positive and negative ion modes. Samples (500 mg per sample) were pretreated and then separated by UHPLC system, followed by mass spectrometry analysis. Samples were mixed in equal amounts to prepare quality control (QC) samples, which were spaced evenly among the injections. All experimental samples were randomly distributed throughout the run in order to monitor the precision and stability of the method during its operation.

The raw data were converted to mzXML files using ProteoWizard MSConvert and processed using XCMS for peak detection, alignment, and data filtering [73, 74]. Mass accuracy tolerance within 25 ppm was used as the mass window for the database search and secondary spectral map matching, and the standards database of the laboratory was retrieved. For the data extracted by XCMS, ion peaks of group sum > 50% were deleted. Pattern recognition was carried out by applying the software SIMCA-P 14.1 (Umetrics, Umea, Sweden).

PLS-DA was performed for the supervised multivariate statistical analysis using MetaboAnalyst (www.metaboanalyst.ca) web-based system. The significantly different metabolites were determined based on the combination of a statistically significant threshold of variable influence on projection (VIP) values obtained from PLS-DA model and two-tailed Student’s t test (*p* value) on the raw data, and the metabolites with VIP values larger than 1.0 and *p* values less than 0.05 were considered as significant. Heat maps were generated using a hierarchical clustering algorithm to visualize the metabolite difference within the data set. The data were normalized using z-scores of the intensity areas of differential metabolites and were ploted by Pheatmap package in R language. The differential metabolites were submitted to the KEGG website (http://www.genome.jp/kegg/pathway.html) for related pathway analysis. The metabolic pathway enrichment of these metabolites was performed by Fisher’s exact test in Omicsbean, when *P*-value of metabolic pathway < 0.05, metabolic pathway was considered as statistically significant enrichment. The metabolomics data were submitted to MetaboLights (https://www.ebi.ac.uk/metabolights/) under accession number MTBLS2089.

The correlation matrix between the gut microflora–related metabolites and gut bacterial species was generated using Pearson’s correlation coefficient.

## Supplementary Information

**Additional file 1 **: **Fig. S1** Chinese monal rarefaction curves (A) and rank abundance curves (B).

## Data Availability

The datasets used and/or analysed during the current study are available from the corresponding author on reasonable request.

## Abbreviations

CITES: Convention on International Trade in Endangered Species of Wild Fauna and Flora
IUCN: International Union for Conservation of Nature
WCM: Wild Chinese monal
CCM: Captive Chinese monal
16S rRNA: 16S ribosomal RNA
UHPLC: Ultra-high performance liquid chromatography
OTU: operational taxonomic unit
PLS-DA: Partial least squares discrimination analysis
NCBI: National Center for Biotechnology Information
BLAST: Basic Local Alignment Search Tool
KEGG: Kyoto Encyclopedia of Genes and Genomes
5-HIAA: 5-hydroxyindoleacetic acid
