# Supplementary figures and images for: Gut microbiota composition and metabolomic profiles of wild and captive Chinese monals (Lophophorus lhuysii)
Source: Front Zool. 2020 Dec 3;17:36. doi: 10.1186/s12983-020-00381-x (PMC7713318; doi:10.1186/s12983-020-00381-x)

A

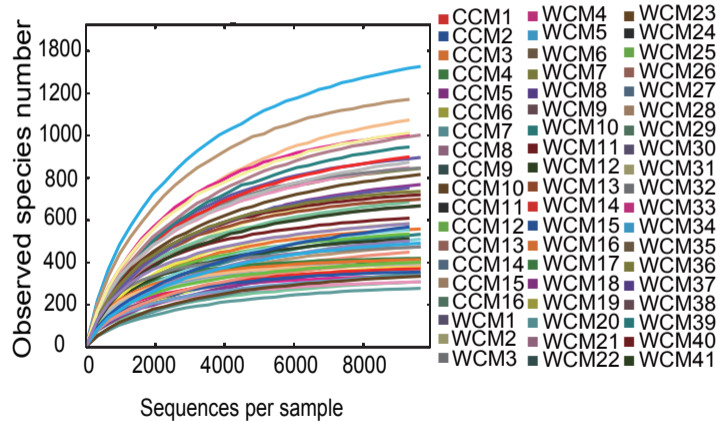

B

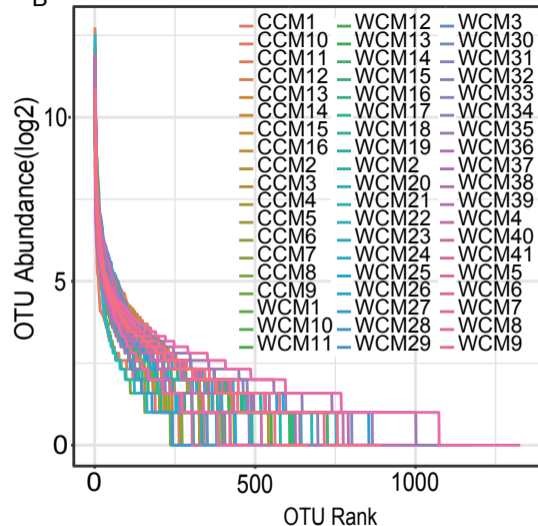

Supplement: Supplementary file 1 — Additional file 1 : Fig. S1 Chinese monal rarefaction curves (A) and rank abundance curves (B). [file 12983_2020_381_MOESM1_ESM.pdf]
